# Antitumor Effect of *Guatteria olivacea* R. E. Fr. (Annonaceae) Leaf Essential Oil in Liver Cancer

**DOI:** 10.3390/molecules27144407

**Published:** 2022-07-09

**Authors:** Alexandre F. C. Galvão, Morgana de S. Araújo, Valdenizia R. Silva, Luciano de S. Santos, Rosane B. Dias, Clarissa A. Gurgel Rocha, Milena B. P. Soares, Felipe M. A. da Silva, Hector H. F. Koolen, Gokhan Zengin, Emmanoel V. Costa, Daniel P. Bezerra

**Affiliations:** 1 Gonçalo Moniz Institute, Oswaldo Cruz Foundation (IGM-FIOCRUZ/BA), Salvador 40296-710, BA, Brazil; alexgalvao10@gmail.com (A.F.C.G.); valdeniziar@gmail.com (V.R.S.); luciano.biomed@gmail.com (L.d.S.S.); rosanebd@gmail.com (R.B.D.); clarissa.gurgel@fiocruz.br (C.A.G.R.); milena.soares@fiocruz.br (M.B.P.S.); 2 Department of Chemistry, Federal University of Amazonas (UFAM), Manaus 69080-900, AM, Brazil; morgana.souz@hotmail.com (M.d.S.A.); felipemas@ufam.edu.br (F.M.A.d.S.); 3 Department of Propedeutics, School of Dentistry, Federal University of Bahia, Salvador 40110-909, BA, Brazil; 4 SENAI Institute for Innovation in Advanced Health Systems, SENAI CIMATEC, Salvador 41650-010, BA, Brazil; 5 Metabolomics and Mass Spectrometry Research Group, Superior School of Health Sciences, Amazonas State University (UEA), Manaus 690065-130, AM, Brazil; hkoolen@uea.edu.br; 6 Department of Biology, Science Faculty, Selcuk University, 42130 Konya, Turkey; gokhanzengin@selcuk.edu.tr

**Keywords:** *Guatteria olivacea*, essential oil, liver cancer, antitumor

## Abstract

*Guatteria olivacea* R. E. Fries (synonym *Guatteria punctata* (Aubl.) R.A. Howard) is a tree of 10–27 m tall popularly known as “envira-bobó”, “envira-fofa”, “envireira”, “embira”, “embira-branca”, “embira-preta”, envira-branca”, and “envira-preta”, which can be found in the Brazilian Amazon biome. In this study, we evaluated the cytotoxic and antitumor effects of the essential oil (EO) obtained from the leaves of *G. olivacea* against liver cancer using HepG2 cells as a model. EO was obtained using a hydrodistillation Clevenger-type apparatus and was qualitatively and quantitatively characterized using GC–MS and GC–FID, respectively. The alamar blue assay was used to assess the cytotoxic potential of EO in a panel of human cancer cell lines and human non-cancerous cells. In HepG2 cells treated with EO, YO-PRO-1/propidium iodide staining, cell cycle distribution, and reactive oxygen species (ROS) were examined. In C.B-17 SCID mice with HepG2 cell xenografts, the efficacy of the EO (20 and 40 mg/kg) was tested in vivo. GC–MS and GC–FID analyses showed germacrene D (17.65%), 1-*epi*-cubenol (13.21%), caryophyllene oxide (12.03%), spathulenol (11.26%), (*E*)-caryophyllene (7.26%), bicyclogermacrene (5.87%), and δ-elemene (4.95%) as the major constituents of *G. olivacea* leaf EO. In vitro cytotoxicity of EO was observed, including anti-liver cancer action with an IC_50_ value of 30.82 μg/mL for HepG2 cells. In HepG2 cells, EO treatment increased apoptotic cells and DNA fragmentation, without changes in ROS levels. Furthermore, the EO inhibited tumor mass in vivo by 32.8–57.9%. These findings suggest that *G. olivacea* leaf EO has anti-liver cancer potential.

## 1. Introduction

Liver cancer is one of the deadliest cancers, with 905,677 newly diagnosed cases and 830,180 deaths worldwide in 2020 [1]. Current liver cancer therapy has serious side effects and tumor resistance, and it can prolong survival by just a few months [2]. Therefore, new therapies are needed.

The family Annonaceae has therapeutic potentials, in which the genus *Guatteria* Ruiz et Pav. is one of the most representative [3,4]. The antitumor potential of the essential oils (EO) from plants belonging to *Guatteria* has been previously explored, including *Guatteria friesiana* (W. A. Rodrigues) Erkens and Maas [5], *Guatteria pogonopus* Mart. [6], *Guatteria australis* A. St.-Hil. [7,8], *Guatteria ferruginea* A. St.-Hil. [8], *Guatteria latifolia* (Mart.) R. E. Fr. [8], *Guatteria sellowiana* Schltdl. [8], *Guatteria blepharophylla* Mart. [9,10], *Guatteria hispida* (R. E. Fr.) Erkens & Maas [9,10], *Guatteria elliptica* R. E. Fr. [11], and *Guatteria megalophylla* Diels [12].

*Guatteria olivacea* R. E. Fr. (synonym *Guatteria punctata* (Aubl.) R. A. Howard) is a tree of 10–27 m tall and of 20–32 cm in diameter, with thick, greenish bark; its flowers are the color of rust. It is found in non-inundated forests, on clayey soil and can be recognized by its leaves that are black to dark brown when dried and by its long-decurrent leaf base. It is popularly known as “envira-bobó”, “envira-fofa”, “envireira”, “embira”, “embira-branca”, “embira-preta”, envira-branca”, and “envira-preta” and can be found in the Brazilian Amazon biome particularly at the states of Amazonas, Acre, and Pará. Its wood is of good quality with wide use in heavy and light construction, furniture and decorative household items, toys, sheets, boxes, and crates. In Suriname, it is used as an edible fruit, and its leaves in herbal baths [13,14,15].

Previous phytochemical investigation on *G. olivacea* reports the isolation and identification of several isoquinoline-derived alkaloids from the bark, including three phenanthrenes, atherosperminine, argentinine, and atherosperminine *N*-oxide; three aporphines, asimilobine, puterine, and discoguattine; two oxoaporphines, liriodenine and oxoputerine; and two tetrahydroprotoberberines, corypalmine and discretine [16]. Moreover, alcoholic extracts from aerial parts of *G. olivacea* showed in vitro antioxidant activity [17]. In addition, the EO from the aerial parts of *G. olivacea* was reported as trypanocidal and antibacterial agent, displaying (*E*)-caryophyllene, germacrene D, *cis*-β-guaiene, δ-cadinene, germacrene B, (*E*)-nerolidol, and spathulenol terpenoids as major chemical constituents [18]. In this work, we aimed to investigate the antitumor potential of *G. olivacea* leaf EO against liver cancer.

## 2. Results and Discussion

### 2.1. Chemical Composition of G. olivacea Leaf EO

The analysis by gas chromatography coupled with mass spectrometry (GC–MS) and flame ionization detector (GC–FID) made it possible to determine the chemical composition of the EO of *G. olivacea* from dry leaves obtained by hydrodistillation in triplicate. EO oil presented a light green coloration with a strong smell, and the yield obtained was 0.18 ± 0.02% in relation to the weight of the dry material of the samples in triplicate. The chemical compounds were identified based on their mass spectra (Figures S1–S13) and their respective retention rates, as well as a comparison with the data available in the literature. After the chemical analysis, a total of thirty-nine compounds were identified, which comprised 96.85% of the EO composition (Table 1). Among the identified compounds, only terpenoids were observed as constituents, from which sesquiterpenes (37 substances) were the dominant class, comprising 94.10% of the EO composition. Monoterpenes (two substances) were also observed but were represented by only 2.75% of the EO sample.

**Figure 1:**
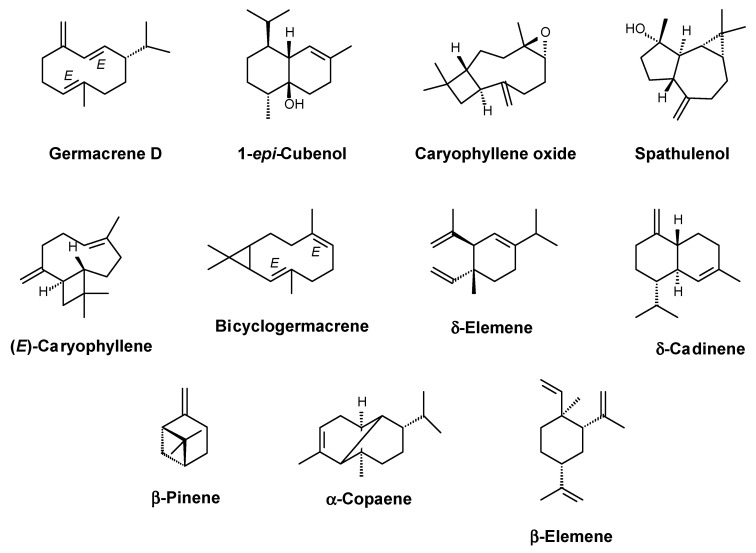
Main compounds identified in *G. olivacea* leaf EO.

The main compounds identified in the EO were germacrene D (17.65%), 1-*epi*-cubenol (13.21%), caryophyllene oxide (12.03%), spathulenol (11.26%), (*E*)-caryophyllene (7.26%), bicyclogermacrene (5.87%), and δ-elemene (4.95%) (Figure 1; Table 1). Other compounds identified with concentrations above 1.4% were: δ-cadinene (2.08%), β-pinene (1.83%), α-copaene (1.69%), and β-elemene (1.48%) (Table 1).

The presence of spathulenol along with some of the major compounds such as germacrene D, caryophyllene oxide, (*E*)-caryophyllene, and bicyclogermacrene was also found in EOs from other *Guatteria* species [6,11,12,19,20]. In fact, particularly spathulenol and caryophyllene oxide together are considered chemophenetic markers of *Guatteria* species EOs [19]. On the other hand, volatile constituents present in this genus displayed significant variations, which could be explained by climatic conditions, geographical localizations, soil characteristics and fertilization level, and seasons, among other factors, which can cause such deviations.

**Table 1:** Chemical composition of *G. olivacea* leaf EO.

Compound		RI ^a^	RI ^b^	Peak Area %
**1**	α-Pinene	930	932	0.92 ± 0.25
**2**	β-Pinene	972	974	1.83 ± 0.38
**3**	δ-Elemene	1335	1335	4.95 ± 0.33
**4**	α-Cubebene	1347	1348	0.45 ± 0.06
**5**	Cyclosativene	1364	1369	0.25 ± 0.03
**6**	α-Ylangene	1368	1373	0.32 ± 0.04
**7**	α-Copaene	1372	1374	1.69 ± 0.18
**8**	β-Bourbonene	1381	1387	0.92 ± 0.09
**9**	β-Cubebene	1387	1387	0.27 ± 0.07
**10**	β-Elemene	1389	1389	1.48 ± 0.14
**11**	Cyperene	1395	1398	0.53 ± 0.06
**12**	α-Gurjunene	1406	1409	1.06 ± 0.10
**13**	(*E*)-Caryophyllene	1415	1417	7.26 ± 0.71
**14**	β-Copaene	1425	1430	0.37 ± 0.10
**15**	γ-Elemene	1431	1434	0.47 ± 0.07
**16**	α-Guaiene	1435	1437	0.26 ± 0.05
**17**	6,9-Guaiadiene	1440	1442	0.23 ± 0.09
**18**	α-Humulene	1450	1452	1.02 ± 0.08
**19**	*allo*-Aromadendrene	1457	1458	0.83 ± 0.06
**20**	γ-Muurolene	1474	1478	0.88 ± 0.12
**21**	Germacrene D	1478	1480	17.65 ± 0.32
**22**	*trans*-Muurol-4(14),5-diene	1488	1493	0.46 ± 0.07
**23**	Bicyclogermacrene	1493	1500	5.87 ± 0.39
**24**	α-Muurolene	1497	1500	0.80 ± 0.07
**25**	δ-Amorphene	1504	1511	0.57 ± 0.04
**26**	γ-Cadinene	1511	1513	0.61 ± 0.06
**27**	δ-Cadinene	1521	1522	2.08 ± 0.23
**28**	*trans*-Cadina-1(2),4-diene	1529	1533	0.30 ± 0.07
**29**	α-Calacorene	1540	1544	0.47 ± 0.09
**30**	Elemol	1546	1548	0.96 ± 0.26
**31**	Germacrene B	1553	1559	1.19 ± 0.14
**32**	Spathulenol	1574	1577	11.26 ± 0.48
**33**	Caryophyllene oxide	1579	1582	12.03 ± 0.95
**34**	Guaiol	1594	1600	0.87 ± 0.01
**35**	Humulene epoxide II	1605	1608	0.69 ± 0.18
**36**	1-*epi*-Cubenol	1618	1627	13.21 ± 0.57
**37**	Cubenol	1639	1645	0.64 ± 0.08
**38**	α-Cadinol	1651	1652	0.80 ± 0.12
**39**	Bulnesol	1664	1670	0.40 ± 0.12
Total monoterpenes				2.75
Total sesquiterpenes				94.10
Total not identified				3.15
Total identified				96.85

RI ^a^ (retention indices) calculated on TR-5MS capillary column (30 m × 0.25 mm × 0.25 µm) according to Van Den Dool and Kratz [21], based on a homologous series of normal alkanes. RI ^b^ (retention indices) according to Adams [22]. Data are presented as mean ± S.D. of three analyses.

### 2.2. In Vitro Cytotoxic Activity of G. olivacea Leaf EO

The in vitro cytotoxic action of *G. olivacea* leaf EO was demonstrated against thirteen cancer cells (HepG2, MCF-7, HCT116, CAL27, HSC-3, SCC-4, KG-1a, HL-60, NB4, THP-1, JURKAT, K562, and B16-F10) and three non-cancerous cells (BJ, MRC-5, and PBMC) for the first time at this study. The IC_50_ values found are shown in Table 2. The EO presented IC_50_ values that ranged from 4.46 to 45.98 μg/mL for the cancer cell lines SCC-4 and K562, respectively. When analyzing cytotoxic activity in non-cancerous cells, EO exhibited IC_50_ values of 47.77 μg/mL for lung fibroblasts (MRC 5) and >50 μg/mL for foreskin fibroblasts (BJ) and mononuclear cells (PBMC). Doxorubicin presented IC_50_ values that ranged from 0.01 to 1.45 μg/mL for the SCC-4 and MCF-7 cancer cell lines, respectively, and 0.91, 0.67, and 0.55 μg/mL for the non-cancerous cells MRC-5, PBMC, and BJ, respectively.

*G. olivacea* leaf EO showed IC_50_ values less than 30 μg/mL for most cell lines tested and was considered promising according to our cytotoxic screening program for new compounds/extracts/oils [5,6,9,10,12,23,24,25]. As a result, it was chosen for further research using the liver cancer HepG2 cells as a cellular model since liver cancer is one of the lethal cancers. These are the first data on the cytotoxicity of *G. olivacea* leaf EO. Interestingly, the EO of *G. friesiana* [5], *G. pogonopus* [6], *G. blepharophylla* [10], *G. hispida* [10], *G. elliptica* [11], and *G. megalophylla* [12] were also previously reported to have IC_50_ values below 30 μg/mL against cancer cell lines.

Cell death is a process that is triggered by a variety of factors and has distinct morphological characteristics depending on the type of cell death, such as apoptosis, necrosis, and autophagy, among others [26]. Apoptosis is the most type of cell death studied, in which this cell death pathway is the target of many cancer treatment strategies. Some features of apoptotic cell death include caspase activation, DNA fragmentation, phosphatidylserine externalization, loss of cell membrane integrity, and PARP cleavage [27]. In this context, the development of drugs with high apoptotic potential is critical.

The apoptotic cell death was quantified using YO-PRO-1/PI-staining HepG2 cells after treatment with EO at concentrations of 12.5, 25, and 50 μg/mL for 48 h. YO-PRO-1 is a nuclear marker that binds to the DNA of dying cells and emits a green fluorescence [28]. Its large size (630 Da) prevents it from penetrating the intact plasma membrane of living cells. However, apoptotic processes compromise membrane integrity, allowing YO-PRO-1 to enter cells, indicating that this dye is an early marker of apoptotic cell death. Its mechanism involves the activation of P2X7 receptors [28], while PI is a nuclear marker that binds to DNA only in dead or damaged cells and emits red fluorescence. As a result, when we use the combination of these two dyes, apoptotic cells show green fluorescence, dead cells (dead cells without identifying the type of cell death) show red and green fluorescence, and viable cells show little or no fluorescence.

**Figure 2:**
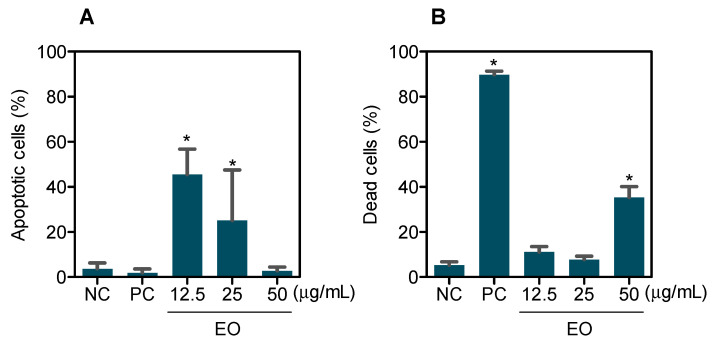
Effect of *G. olivacea* leaf EO on apoptosis induction in HepG2 cells after 48 h of treatment. (**A**) Quantification of apoptotic cells. (**B**) Quantification of dead cells (dead cells without identifying the type of cell death). Negative control (**NC**) was treated with vehicle (0.5% DMSO) used for diluting EO, and doxorubicin (0.5 µg/mL) was used as positive control (**PC**). Data are presented as mean ± S.E.M. of at least three independent experiments. * *p* < 0.05 compared with negative control by ANOVA, followed by Student–Newman–Keuls test.

As shown in Figure 2, a statistically significant increase in the percentage of cells undergoing apoptosis was found in HepG2 cells treated with EO at concentrations of 12.5 and 25 μg/mL after 48 h of treatment, while the increase in dead cells was observed at a concentration of 50 μg/mL. Doxorubicin, used as a positive control, significantly increased the percentage of dead cells.

**Figure 3:**
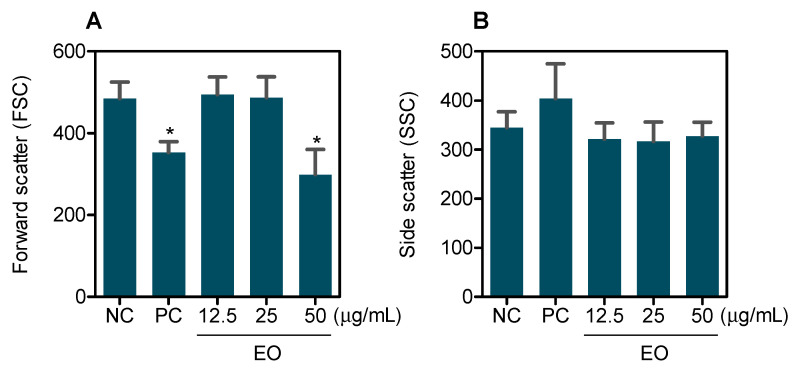
Effect of *G. olivacea* leaf EO in HepG2 cell morphology, as determined by light-scattering features detected by flow cytometry after 48 h of treatment. (**A**) Forward scatter. (**B**) Side scatter. Negative control (**NC**) was treated with vehicle (0.5% DMSO) used for diluting EO, and doxorubicin (0.5 µg/mL) was used as positive control (**PC**). Data are presented as mean ± S.E.M. of at least three independent experiments. * *p* < 0.05 compared with negative control by ANOVA, followed by Student–Newman–Keuls test.

Furthermore, EO caused cell shrinkage, as measured by a decrease in forwarding light scatter, a morphological change seen in apoptotic cells (Figure 3). Measurement of ROS levels was also performed after treatment of HepG2 cells with EO at the same concentrations for 1 and 3 h; however, no significant changes were observed (data not shown).

**Figure 4:**
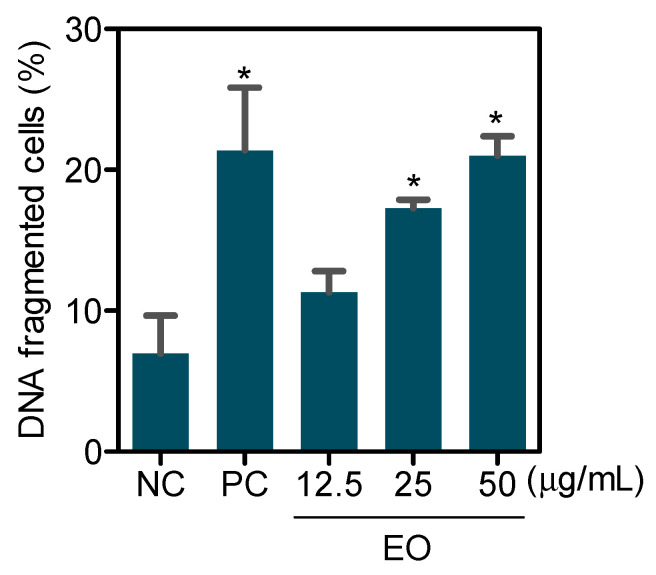
Effect of *G. olivacea* leaf EO in the DNA fragmentation of HepG2 cells after 48 h of treatment. Negative control (**NC**) was treated with vehicle (0.5% DMSO) used for diluting EO, and doxorubicin (0.5 µg/mL) was used as positive control (**PC**). Data are presented as mean ± S.E.M. of three independent experiments performed in duplicate. * *p* < 0.05 compared with negative control by ANOVA, followed by Student–Newman–Keuls test.

Cell cycle phases and internucleosomal DNA fragmentation were also quantified in HepG2 cells treated with EO at concentrations of 12.5, 25, and 50 μg/mL for 48 h. A significant increase in cells with fragmented DNA was observed after treatment with EO at concentrations of 25 and 50 μg/mL (Figure 4). Doxorubicin, used as a positive control, caused G_2_/M arrest and significantly increased cells with fragmented DNA.

As mentioned above, the cytotoxicity of some EOs from the genus *Guatteria* has previously been reported in different histological types of cancer [5,6,7,8,9,10,11,12]. Among them, HepG2 cells treated with EOs obtained of *G. blepharophylla* and *G. hispida* caused cell morphologies consistent with apoptosis, as well as a remarkable activation of caspase-3 and DNA fragmentation without changes in cell membrane integrity [10]. Similarly, the EO of *G. megalophylla* increased phosphatidylserine externalization and DNA fragmentation in the leukemia HL-60 cells [12].

Notably, some of the major constituents of *G. olivacea* leaf EO have been reported to be cytotoxic. δ-Elemene, a sesquiterpene hydrocarbon, has been shown to induce cell death in human lung carcinoma cells by inhibiting the NF-kB pathway [29]. (*E*)-Caryophyllene, another sesquiterpene hydrocarbon, caused cytotoxicity in human lung cancer cells by causing G_1_ cell cycle arrest [30]. Germacrene D and bicyclogermacrene, other sesquiterpene hydrocarbons, have also been reported as cytotoxic agents against melanoma, leukemia, and colon cancer [31,32]. Caryophyllene oxide, a sesquiterpene oxygenated, caused apoptotic cell death in prostate cancer cells, as evidenced by depolarization of the mitochondrial membrane, morphological changes, and caspases activation [33]. Spathulenol, another sesquiterpene oxygenated, has been shown to be cytotoxic against melanoma, leukemia, and liver cancer cell lines [34].

### 2.3. In Vivo Antitumor Effect of G. olivacea Leaf EO

**Figure 5:**
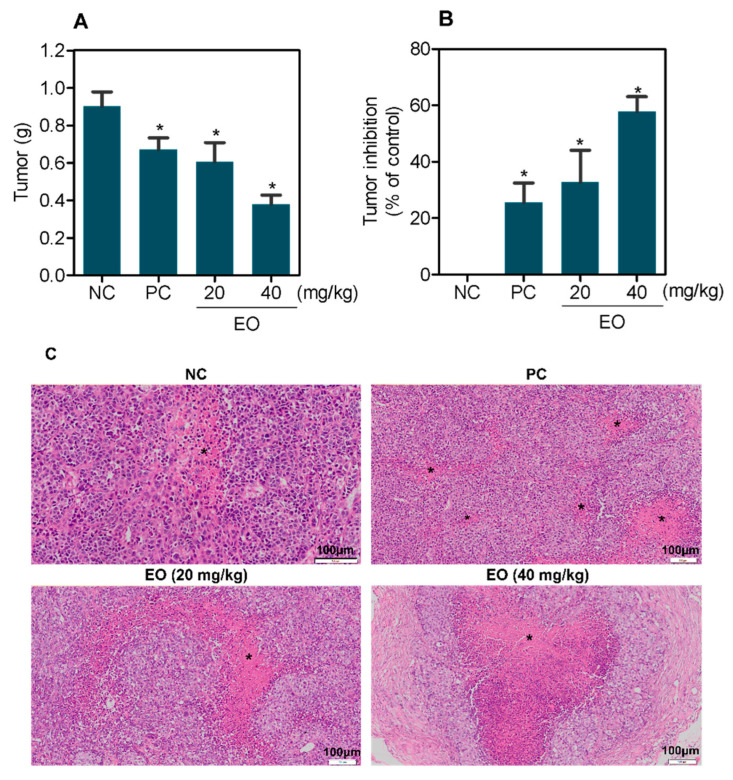
In vivo anti-liver cancer effect of *G. olivacea* leaf EO in C.B-17 SCID mice with HepG2 cell xenografts. (**A**) Tumor weight (g) after treatment. (**B**) Tumor inhibition (%) after treatment. (**C**) Representative photomicrographs of HepG2 tumors, stained with hematoxylin and eosin and analyzed by light microscopy. Asterisks represent areas of tissue necrosis. Negative control (**NC**) was treated with vehicle (5% DMSO) used for diluting EO, and doxorubicin (0.8 mg/kg) was used as positive control (**PC**). Data are presented as mean ± S.E.M. of 8–17 animals. * *p* < 0.05 compared with negative control by ANOVA, followed by Student–Newman–Keuls test.

The antitumor activity of *G. olivacea* leaf EO was studied in CB-17 SCID mice inoculated with HepG2 cells. The animals were treated intraperitoneally with doses of 20 and 40 mg/kg of EO once a day for 21 days (Figure 5). At the end of treatment, the mean weight of tumors in negative control animals was 0.90 ± 0.07 g. The mean tumor weights in EO-treated animals were 0.38 ± 0.14 and 0.61 ± 0.10 g at 40 and 20 mg/kg doses, respectively. Tumor mass inhibition was 57.9 and 32.8%, respectively. Doxorubicin reduced tumor mass by 25.6%. The H&E-stained tumors showed a collection of proliferative hyperchromatic malignant cells exhibiting anisocytosis. Mitotic events were frequent in both groups, some atypical. In animals treated with EO at a dose of 40 mg/kg, tumor nodules were smaller and exhibited a denser surrounding connective tissue when compared to negative control. Necrotic areas were observed in all groups, especially in doxorubicin-treated mice.

**Figure 6:**
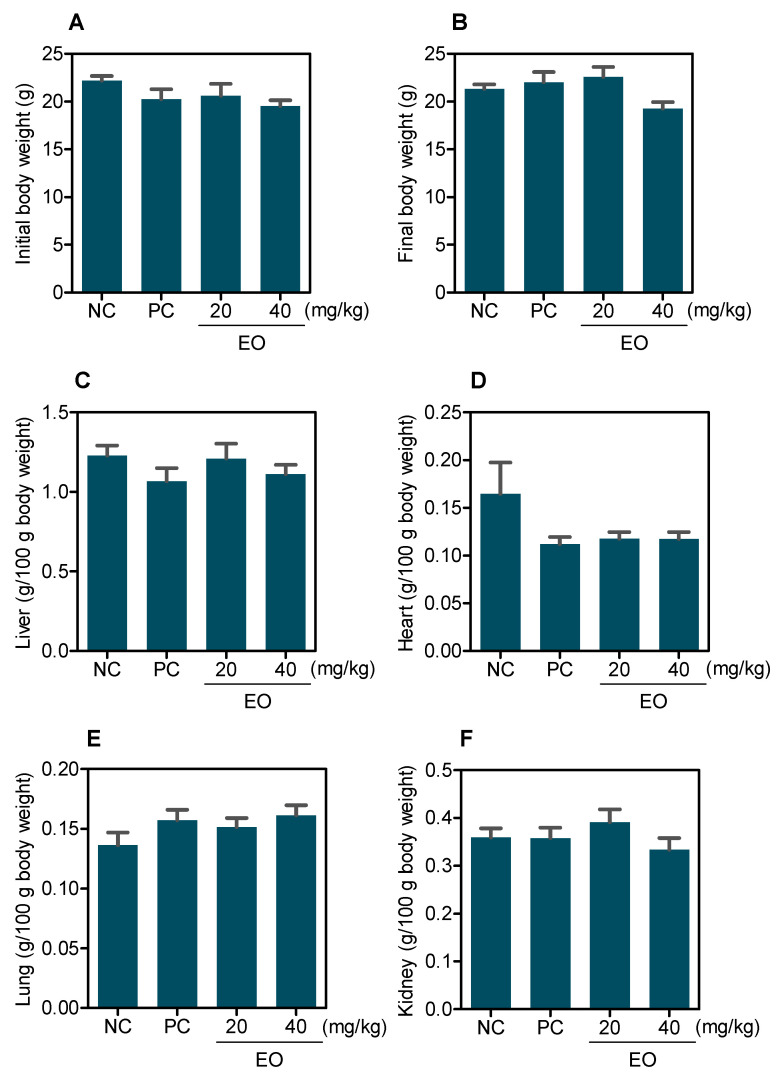
Effect of *G. olivacea* leaf EO on body and relative organ weight from C.B-17 SCID mice with HepG2 cell xenografts. (**A**) Initial body weight. (**B**) Final body weight. (**C**) Liver. (**D**) Heart. (**E**) Lung. (**F**) Kidney. Negative control (**NC**) was treated with vehicle (5% DMSO) used for diluting EO, and doxorubicin (0.8 mg/kg) was used as positive control (**PC**). Data are presented as mean ± S.E.M. of 8–17 animals.

All groups had 100% survival rates, and no significant changes in body and organ weights (liver, kidney, lung, and heart) were observed in any group (*p* > 0.05) (Figure 6).

Histopathological analyses of the liver, kidney, lung, and heart were also performed. The livers of all experimental groups presented a preserved portal architecture. In general, some morphological changes were observed, such as vascular congestion ranging from mild to moderate, mild hydropic degeneration, and coagulation necrosis in focal areas of the organ. Isolated inflammatory cells were observed around the portal system, predominantly polymorphonuclear cells. Importantly, only the animals treated with EO at dose of 40 mg/kg showed moderate microgoticular steatosis (Figure S14). The renal architecture was preserved in all animals in the present study. However, some histopathological changes were observed, such as vascular congestion ranging from moderate to intense and a decrease in the urinary space (Bowman’s space) by glomerular hyalinization. Focal areas of coagulation necrosis in the tubules of the renal cortex were observed in doxorubicin- and EO-treated groups (Figure S15). The architecture of the lung parenchyma ranged from preserved to partially preserved in this study. The histopathological changes observed were vascular congestion, edema, and thickening of the interalveolar septa with reduced alveolar lumen, which ranged from mild to intense. In addition, focal areas of hemorrhage, fibrosis, and inflammatory cells, predominantly polymorphonuclear, were observed in all experimental groups (Figure S16). For the hearts, we did not observe any noteworthy architectural and morphological changes for this organ.

Likewise, *G. megalophylla* leaf EO inhibited tumor mass by 16.63% at 50 mg/kg and 48.79% at 100 mg/kg in mice engrafted with HL-60 cells, with no significant change in the relative weight of organs from any of the groups studied [12]. The antitumor effect of *G. friesiana* leaf EO was studied in a murine model using S180-bearing mice. When administered intraperitoneally (50 and 100 mg/kg) and orally (100 and 200 mg/kg), the tumor growth inhibition rates were 43.4–54.2% and 6.6–42.8%, respectively [5]. *G. pogonopus* leaf EO also inhibited S180 tumor growth by 25.3–42.6% when administered intraperitoneally at 50 and 100 mg/kg, respectively [6].

In conclusion, *G. olivacea* leaf EO has in vitro and in vivo anti-liver cancer activity, which can be associated with the mixture of its main constituents sesquiterpene hydrocarbons, (*E*)-caryophyllene, bicyclogermacrene, δ-elemene, and germacrene D and oxygenated sesquiterpenes, caryophyllene oxide, 1-*epi*-cubenol, and spathulenol.

## 3. Materials and Methods

### 3.1. Botanical Material

Leaves of *G. olivacea* (15–18 × 4.5–5.5 cm) were collected on 27 May 2017 on the Adolpho Ducke Reserve, (geographic coordinates: 2°54′ 47″ S and 59°58′ 48″ W), Manaus, Amazonas State, Brazil, and identified by Prof. Antonio Carlos Webber, a plant taxonomist of the Department of Biology of the Federal University of Amazonas (DB/UFAM). A voucher specimen number 11,423 was deposited at the Herbarium of DB/UFAM. The access (specimen) was registered in the Sistema Nacional de Gestão do Patrimônio Genético e do Conhecimento Tradicional Associado (SISGEN) with the record A70EDCD.

### 3.2. Chemical Evaluation

#### 3.2.1. Essential Oil Extraction

The leaves of *G. olivacea* were oven-dried with air circulation at 40 °C for 24 h and subjected to hydrodistillation (900 g) for 4 h using a Clevenger-type apparatus (Amitel, São Paulo, Brazil). Hydrodistillation was performed in triplicate (3 × 300 g = 900 g). The essential oil samples (in triplicate) were dried with anhydrous sodium sulfate (Na_2_SO_4_), and the percentage of their content was calculated based on the weight of the dry material used in each hydrodistillation. Subsequently, the standard deviation of the triplicate was calculated. EOs were stored in the freezer prior to chemical and biological analyses.

#### 3.2.2. GC–FID and GC–MS Analyses

The GC-FID analysis was carried out using a Shimadzu GC-17A GC system equipped with a DB-5MS capillary column (30 m × 0.25 mm × 0.25 µm). Helium was the carrier gas at 1 mL/min flow rate. The injection solution was prepared by dissolving about 10 mg of oil in 1 mL of dichloromethane, being 1 μL of this solution injected at a split ratio of 1:50. The column temperature program was: 40 °C/4min, a rate of 4 °C/min to 240 °C, then a rate of 10 °C/min to 280 °C, and then 280 °C/2 min [35]. The injection and detector temperatures were 250 and 220 °C, respectively. The GC-MS analysis was carried out with a Trace Ultra gas chromatograph coupled to an ISQ single quadrupole mass spectrometer (Thermo Scientific). This system was equipped with a Tri Plus autosampler and a TR-5MS capillary column (30 m × 0.25 mm × 0.25 µm). The injection, interface, and ion source temperatures were 250, 250, and 220 °C, respectively. Mass spectrometry acquisitions were performed at a mass range of *m*/*z* 40–440. The other conditions were the same as those used for GC analysis.

The identifications of the oil components were performed based on a comparison of the obtained mass spectra with those stored in the NIST library and also by comparison of retention indexes (RI) with published data [22]. To obtain the RI, a homologous series of linear hydrocarbons (C_8_–C_20_) was injected under the same analysis conditions, and the calculations were performed according to the Van den Dool and Kratz equation [21].

### 3.3. Pharmacological Evaluation

#### 3.3.1. Alamar Blue Assay

HepG2 (human hepatocellular carcinoma), MCF-7 (human breast adenocarcinoma), HCT116 (human colon carcinoma), CAL27 (human oral squamous cell carcinoma), HSC-3 (human oral squamous cell carcinoma), SCC-4 (human oral squamous cell carcinoma), KG-1a (human myeloid leukemia), HL-60 (human acute promyelocytic leukemia), NB4 (human acute promyelocytic leukemia), THP-1 (human monocytic leukemia), JURKAT (human acute T cell leukemia), K562 (human chronic myelogenous leukemia), B16-F10 (mouse melanoma), BJ (human foreskin fibroblast), and MRC-5 (human lung fibroblast) cell lines were obtained from the American Type Culture Collection (ATCC, Manassas, VA, USA) and cultured in accordance with the ATCC animal cell culture guide. To validate the use of contamination-free cells, all cell lines were tested for mycoplasma using a mycoplasma stain kit (Sigma-Aldrich Co., Saint Luis, MO, USA).

The standard ficoll density protocol was used to obtain primary cell culture of peripheral blood mononuclear cells (PBMC) from healthy donors’ peripheral blood. PBMC were resuspended in RPMI 1640 medium containing 20% fetal bovine serum and 50 μg/mL gentamicin and maintained at 37 °C with 5% CO_2_. Concanavalin A (10 μg/mL, Sigma-Aldrich Co., Saint Luis, MO, USA) was added at the start of the culture as a mitogen to trigger cell division in T lymphocytes. The experimental protocol (number #031019/2013) was approved by the Oswaldo Cruz Foundation’s Research Ethics Committee in Salvador, Bahia, Brazil.

The cell viability was determined using the Alamar blue assay, as previously described [36,37,38]. Cells were grown in 96-well plates for all experiments. EO was dissolved in dimethyl sulfoxide (DMSO, Vetec Qumica Fina Ltd.a, Duque de Caxias, RJ, Brazil) and added to each well before incubating for 72 h. Doxorubicin (doxorubicin hydrochloride, purity 95%, Laboratory IMA S.A.I.C., Buenos Aires, Argentina) was used as a positive control. At the end of the treatment, 20 μL of resazurin stock solution (0.312 mg/mL) (Sigma-Aldrich Co., Saint Luis, MO, USA) was added to each well. SpectraMax 190 Microplate Reader was used to measure absorbances at 570 and 600 nm (Molecular Devices, Sunnyvale, CA, USA).

#### 3.3.2. Flow Cytometry Assays

Apoptotic cells were quantified using YO-PRO-1 (Sigma-Aldrich Co.) and propidium iodide (PI) (BD Biosciences, San Jose, CA, USA) dyes [39]. Briefly, cells were stained with a solution containing 0.1 µM YO-PRO-1 and 1.5 µM PI, and cell fluorescence was determined by flow cytometry.

Quantification of DNA content was used to assess internucleosomal DNA fragmentation and cell cycle distribution [40]. Cells were harvested in a permeabilization solution containing 0.1% triton X-100, 2 μg/mL PI, 0.1% sodium citrate, and 100 μg/mL RNAse (all from Sigma-Aldrich Co.), and flow cytometry was used to determine cell fluorescence.

At least 10^4^ events were recorded per sample for all flow cytometry analyses. A BD LSRFortessa cytometer was used along with BD FACSDiva Software (BD Biosciences) and FlowJo Software 10 (FlowJo Lcc; Ashland, OR, USA). Cellular debris was omitted from the analysis.

#### 3.3.3. Reactive Oxygen Species Quantification

2′,7′-dichlorofluorescin diacetate (DCF-DA) (Sigma-Aldrich Co., Saint Luis, MO, USA) was used to measure intracellular reactive oxygen species (ROS) [41]. Cells were collected, washed with saline, and stained for 30 min with 5 μM DCF-DA. The cells were then washed with saline, and cell fluorescence was measured using a Fluoroskan™ microplate fluorometer (Thermo Scientific™, Waltham, MA, USA).

#### 3.3.4. Human Liver Cancer Xenograft Model

A total of 43 C.B-17 severe combined immunodeficient (SCID) mice (males and females, six weeks old, 20–25 g) were obtained and kept at the Gonçalo Moniz Institute-FIOCRUZ animal facilities (Salvador, Bahia, Brazil). The animals were kept in cages with free access to food and water. All animals were subjected to a 12:12 h light–dark cycle (lights on at 7:00 a.m.). The experimental protocol was approved by a local animal ethics committee (number #01/2021).

HepG2 cells (10^7^ cells/500 µL/mouse) were implanted subcutaneously into the left front armpit, as previously described [23,24,25]. After one day, the animals were treated intraperitoneally (200 μL/mouse) once a day for 21 days. The mice were divided into four groups: #1 injected with vehicle (5% DMSO solution) used for diluting EO (*n* = 17); #2 injected with doxorubicin (0.8 mg/kg, *n* = 8); #3 injected with EO at 20 mg/kg (n = 9); and #4 injections with EO at 40 mg/kg (*n* = 9). One day after the end of treatment, an anesthetic overdose (thiopental, 100 mg/kg) was used to euthanize the animals, and tumors were excised and weighed. The inhibition ratio (percent) was calculated using the following formula: inhibition ratio (percent) = [(A − B)/A] × 100, where A is the average tumor weight of the negative control and B is the tumor weight of the treated group.

To assess toxicological effects, the mice were weighed at the start and end of the experiment. Throughout the study, animals were monitored for abnormalities. The tumor, livers, kidneys, lungs, and hearts were excised and weighed. Macroscopically, the organs were analyzed for the presence of metastatic nodules, color changes, and hemorrhage. Histological analyses for tumors and organs were performed under optical microscopy using hematoxylin-eosin and periodic acid–Schiff (liver and kidney) staining after fixation in 4% formaldehyde.

### 3.4. Statistical Analysis

The data were presented as mean ± S.E.M. or as the half-maximal inhibitory concentration (IC_50_) value with 95% confidence intervals derived from nonlinear regressions from at least three independent experiments performed in duplicate. ANOVA was used to compare differences between experimental groups, followed by Student–Newman–Keuls test (*p* < 0.05). GraphPad Prism was used to perform all statistical analyses (Intuitive Software for Science; San Diego, CA, USA).

## Figures and Tables

**Table 2:** Cytotoxic activity of *G. olivacea* leaf EO.

Cells	Histological Type	IC_50_ and 95% CI (in μg/mL)	
		DOX	EO
**Cancer cells**			
HepG2	Human hepatocellular carcinoma	0.09 0.06–0.12	30.82 20.57–46.18
MCF-7	Human breast adenocarcinoma	1.45 1.00–2.11	22.03 14.17–34.26
HCT116	Human colon carcinoma	0.06 0.03–0.12	24.11 19.75–29.44
CAL27	Human oral squamous cell carcinoma	0.65 0.26–1.65	32.23 19.75–52.59
HSC-3	Human oral squamous cell carcinoma	0.66 0.49–0.87	30.06 22.00–41.07
SCC-4	Human oral squamous cell carcinoma	0.01 0.002–0.04	4.46 4.03–4.95
KG-1a	Human myeloid leukemia	0.01 0.01–0.11	26.75 23.34–30.67
HL-60	Human acute promyelocytic leukemia	0.05 0.03–0.10	23.46 12.88–42.73
NB4	Human acute promyelocytic leukemia	0.05 0.03–0.07	33.65 31.51–35.92
THP-1	Human monocytic leukemia	0.08 0.05–0.12	36.93 29.93–45.57
JURKAT	Human acute T cell leukemia	0.03 0.02–0.05	26.44 24.12–28.98
K562	Human chronic myelogenous leukemia	0.70 0.36–1.36	45.98 38.74–54.57
B16-F10	Mouse melanoma	0.28 0.23–0.35	28.30 20.93–38.26
**Non-cancerous cells**			
BJ	Human foreskin fibroblast	0.55 0.22–1.37	>50
MRC-5	Human lung fibroblast	0.91 0.30–2.73	47.77 35.76–63.81
PBMC	Human peripheral blood mononuclear cells	0.67 0.48–0.94	>50

Doxorubicin (DOX) was used as a positive control.

## Data Availability

Not applicable.

## Supplementary Materials

The following supporting information can be downloaded at: https://www.mdpi.com/article/10.3390/molecules27144407/s1. Mass spectra and histological data of liver, lung and kidney (Figures S1–S16). Figure S1. (A) Chromatogram of the total ions of *G. olivacea* leaf EO (triplicate); (B) Enlargement of the region between 22.0 min to 36.0 min. Figure S2. Mass spectrum of α-pinene (t_R_ 9.27 min). Figure S3. Mass spectrum of β-pinene (t_R_ 10.89 min). Figure S4. Mass spectrum of δ-elemene (t_R_ 24.15 min). Figure S5. Mass spectrum of β-elemene (t_R_ 25.92 min). Figure S6. Mass spectrum of (*E*)-caryophyllene (t_R_ 26.75 min). Figure S7. Mass spectrum of α-humulene (t_R_ 27.81 min). Figure S8. Mass spectrum of germacrene D (t_R_ 28.67 min). Figure S9. Mass spectrum of bicyclogermacrene (t_R_ 29.13 min). Figure S10. Mass spectrum of δ-cadinene (t_R_ 29.96 min). Figure S11. Mass spectrum of spathulenol (t_R_ 31.51 min). Figure S12. Mass spectrum of caryophyllene oxide (t_R_ 31.64 min). Figure S13. Mass spectrum of 1-epi-cubenol (t_R_ 32.98 min). Figure S14. Representative photomicrographs of the livers of the C.B-17 SCID mice with HepgG2 cell xenografts treated with *G. olivacea* leaf EO. Negative control (NC) was treated with vehicle (5% DMSO) used for diluting EO, and doxorubicin (0.8 mg/kg) was used as positive control (PC). Figure S15. Representative photomicrographs of the lungs of the C.B-17 SCID mice with HepgG2 cell xenografts treated with *G. olivacea* leaf EO. Negative control (NC) was treated with vehicle (5% DMSO) used for diluting EO, and doxorubicin (0.8 mg/kg) was used as positive control (PC). Figure S16. Representative photomicrographs of the kidneys of the C.B-17 SCID mice with HepgG2 cell xenografts treated with *G. olivacea* leaf EO. Negative control (NC) was treated with vehicle (5% DMSO) used for diluting EO, and doxorubicin (0.8 mg/kg) was used as positive control (PC).

## Sample Availability

Sample of the EO is not available from the authors.
