# Supplementary material for: Antitumor Effect of Guatteria olivacea R. E. Fr. (Annonaceae) Leaf Essential Oil in Liver Cancer
Source: Molecules. 2022 Jul 9;27(14):4407. doi: 10.3390/molecules27144407 (PMC9319081; doi:10.3390/molecules27144407)
Supplement: Supplementary file 1 [file molecules-27-04407-s001.zip › molecules-1791482-supplementary.pdf]

Supplementary Material

Antitumor effect of *Guatteria olivacea* R. E. Fr. (Annonaceae) leaf essential oil in liver cancer

Alexandre F. C. Galvão[1], Morgana de S. Araújo[2], Valdenizia R. Silva[1], Luciano de S. Santos[1], Rosane B. Dias[1,3], Clarissa A. G. Rocha[1,3], Milena B. P. Soares[1,4], Felipe M. A. da Silva[2], Hector H. F. Koolen[5], Gokhan Zengin[6], Emmanoel V. Costa[2,*], Daniel P. Bezerra[1,*]

[1]Gonçalo Moniz Institute, Oswaldo Cruz Foundation (IGM-FIOCRUZ/BA), Salvador, BA, 40296-710, Brazil.

[2]Department of Chemistry, Federal University of Amazonas (UFAM), Manaus, AM, 69080-900, Brazil.

[3]Department of Propedeutics, School of Dentistry of the Federal University of Bahia, Salvador 40110-909, BA, Brazil.

[4]SENAI Institute for Innovation in Advanced Health Systems, SENAI CIMATEC, Salvador, BA, 41650-010, Brazil.

[5]Metabolomics and Mass Spectrometry Research Group, Superior School of Health Sciences, Amazonas State University (UEA), Manaus, AM, 690065-130, Brazil.

[6]Department of Biology, Science Faculty, Selcuk University, 42130, Konya, Turkey.

*Corresponding authors:

E. V. Costa (e-mail: emmanoelvc@gmail.com); D. P. Bezerra (e-mail: daniel.bezerra@fiocruz.br); Tel./Fax: +55-92-3305-1181 Ramal 2870 (E.V. Costa); Tel./Fax: +55-71-3176-2272 (D. P. Bezerra).

A

B

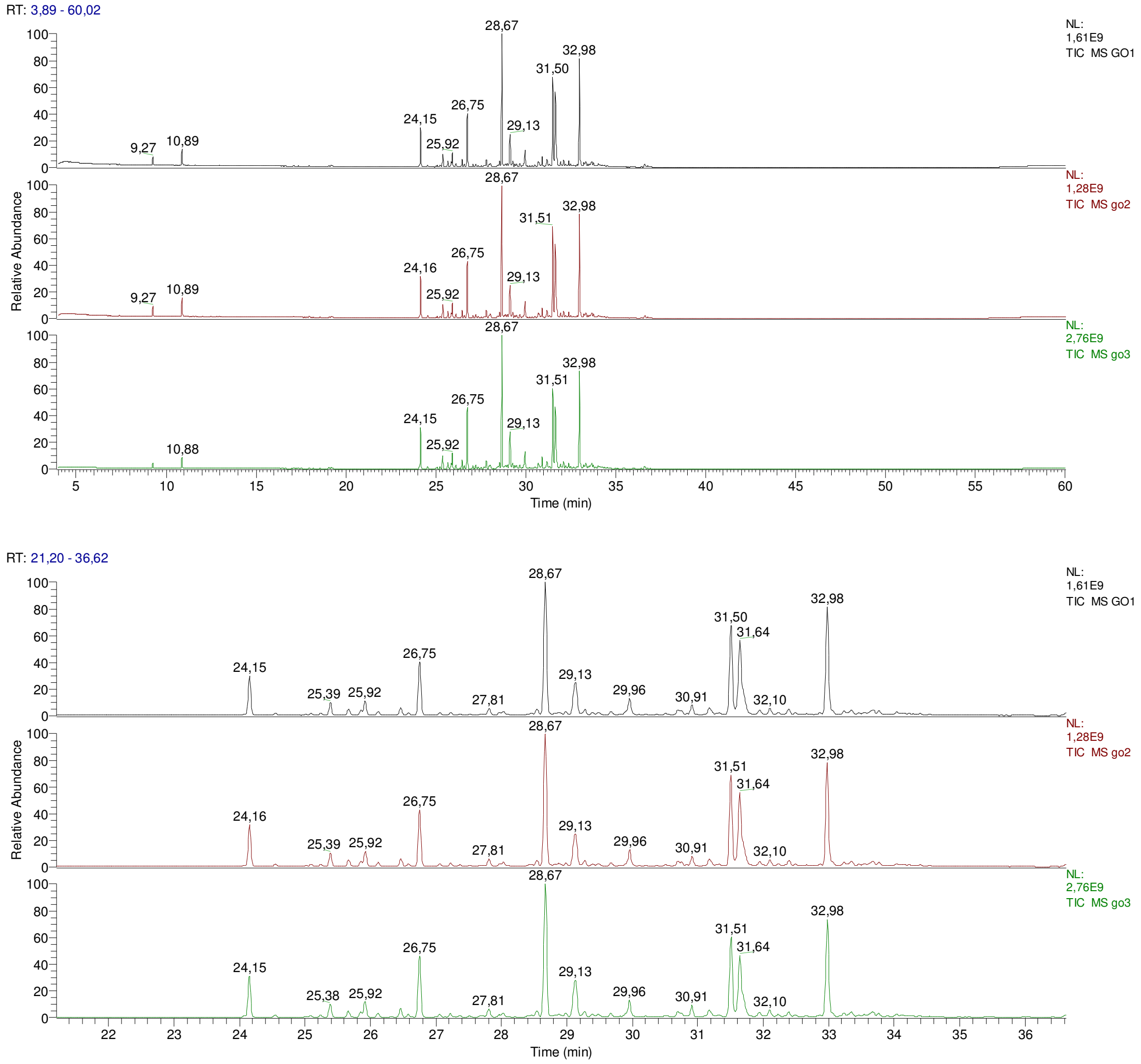

Figure S1. (**A**) Chromatogram of the total ions of *G. olivacea* leaf EO (triplicate); (**B**) Enlargement of the region between 22.0 min to 36.0 min.

Retention time of some main constituents present in the OE:

**α-Pinene**<br>(9.27 min)	**β-Pinene**<br>(10.89 min)	**δ-Elemene**<br>(24.15; 24.16 min)	**β-Elemene**<br>(25.92 min)
**(*E*)-Caryophyllene**<br>(26.75 min)	**α-Humulene**<br>(27.81 min)	**Germacrene D**<br>(28.67 min)	**Bicyclogermacrene**<br>(29.13 min)
**δ-Cadinene**<br>(29.96 min)	**Spathulenol**<br>(31.50; 31.51 min)	**Caryophyllene oxide**<br>(31.64 min)	**1-*epi*-Cubenol**<br>(32.98 min)

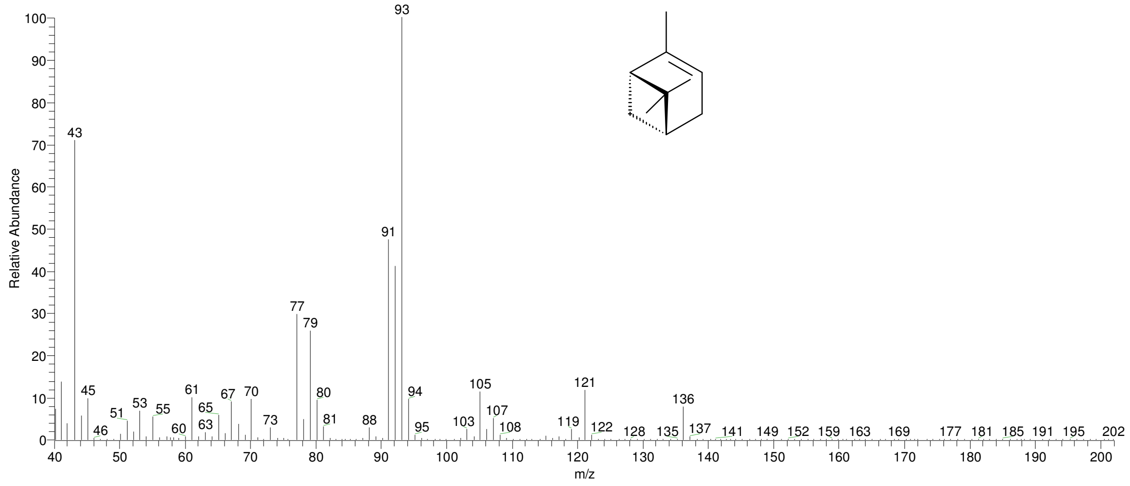

Figure S2. Mass spectrum of α-pinene (t_R 9.27 min).

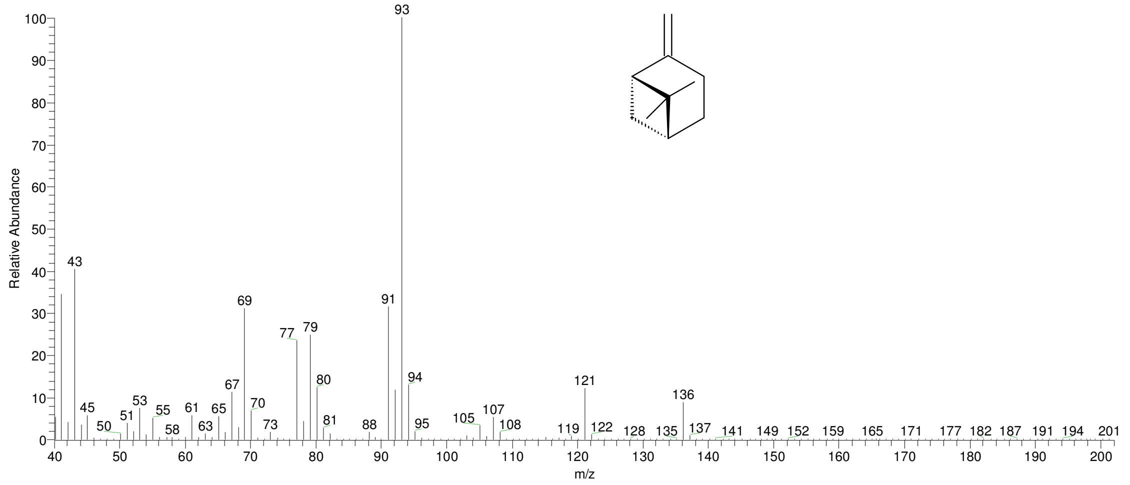

Figure S3. Mass spectrum of β-pinene (t_R 10.89 min).

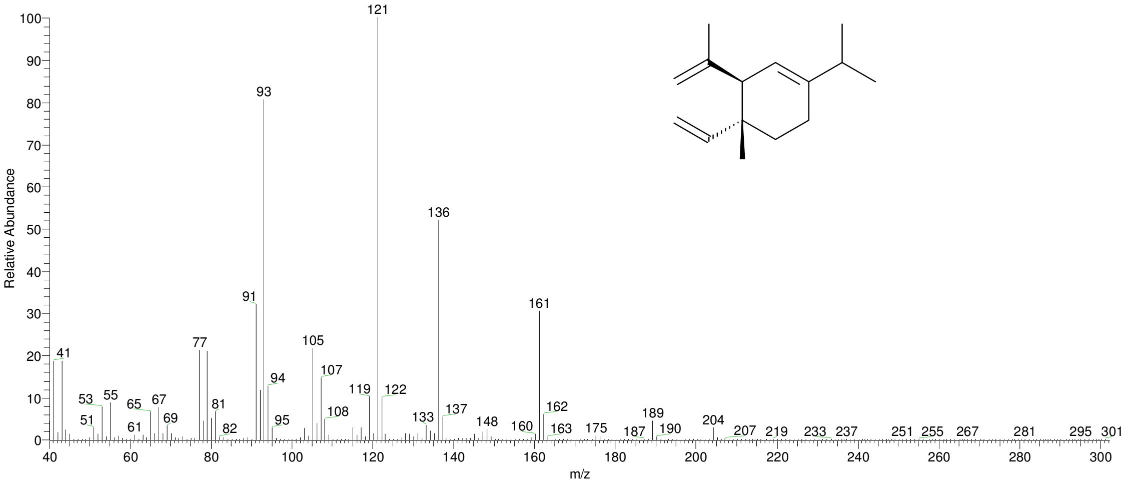

Figure S4. Mass spectrum of δ-elemene (t_R 24.15 min).

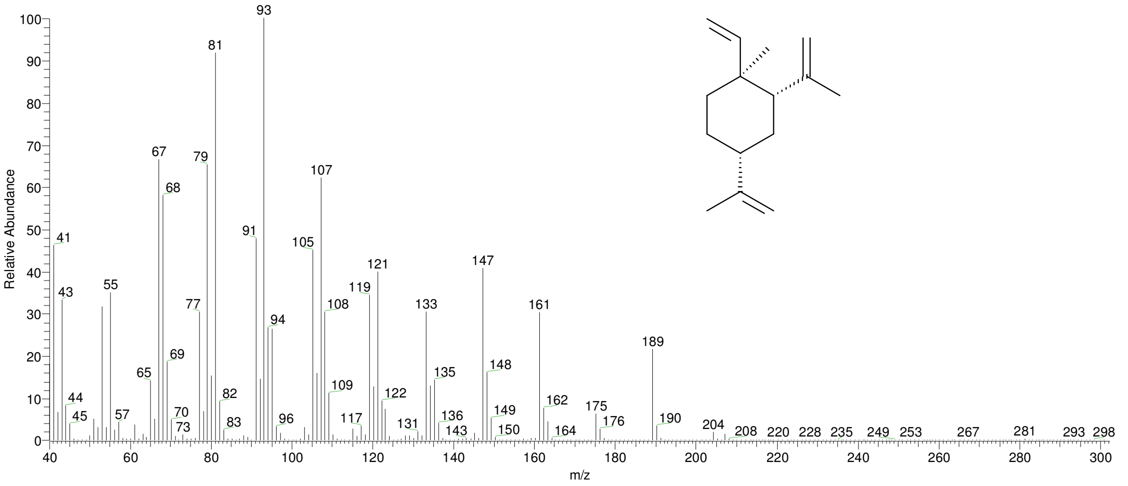

Figure S5. Mass spectrum of β-elemene (t_R 25.92 min).

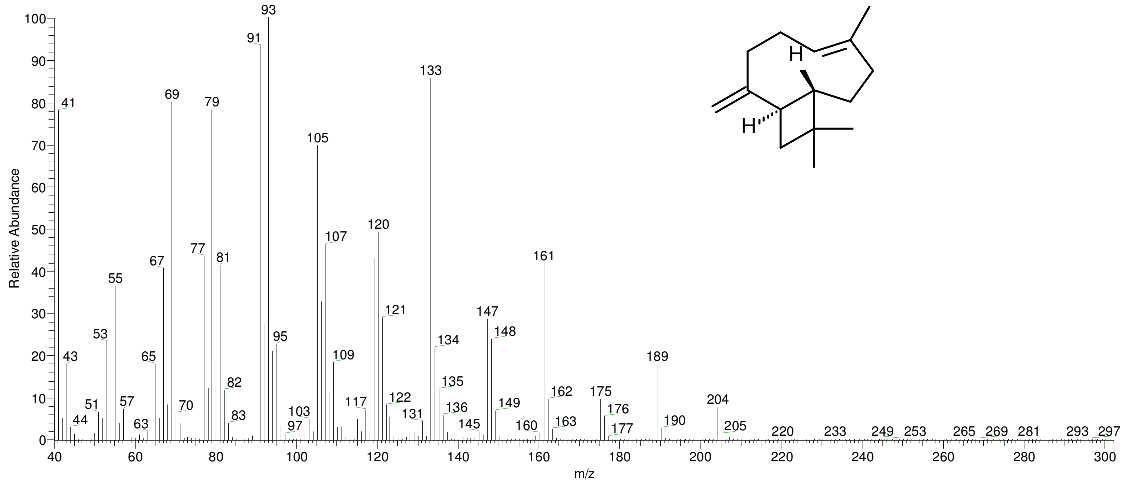

Figure S6. Mass spectrum of (*E*)-caryophyllene (t_R 26.75 min).

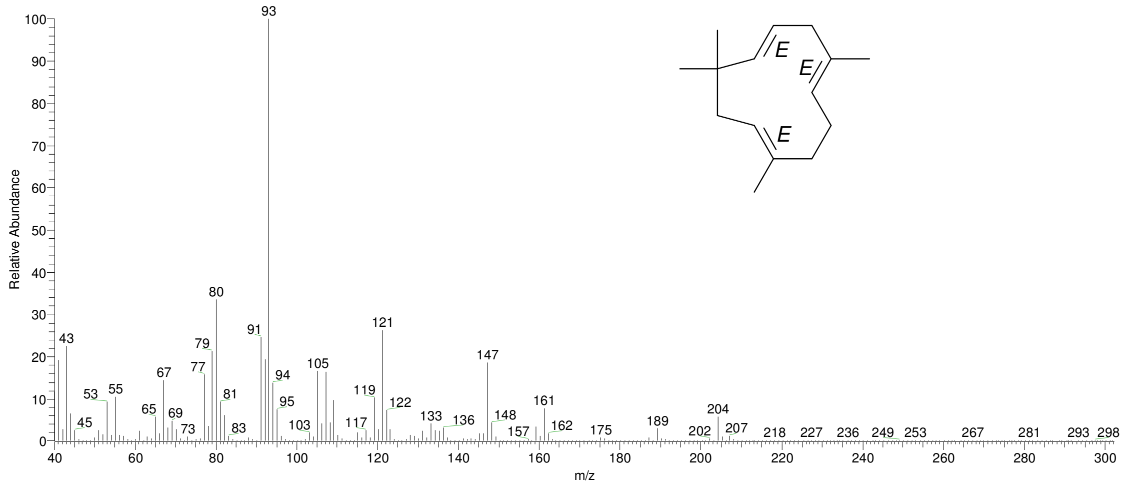

Figure S7. Mass spectrum of α-humulene (t_R 27.81 min).

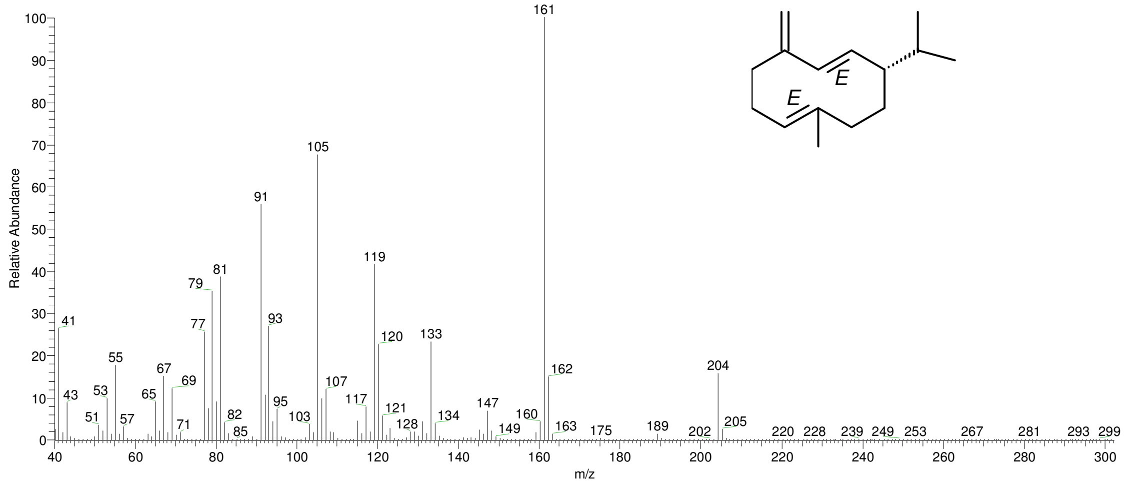

Figure S8. Mass spectrum of germacrene D (t_R 28.67 min).

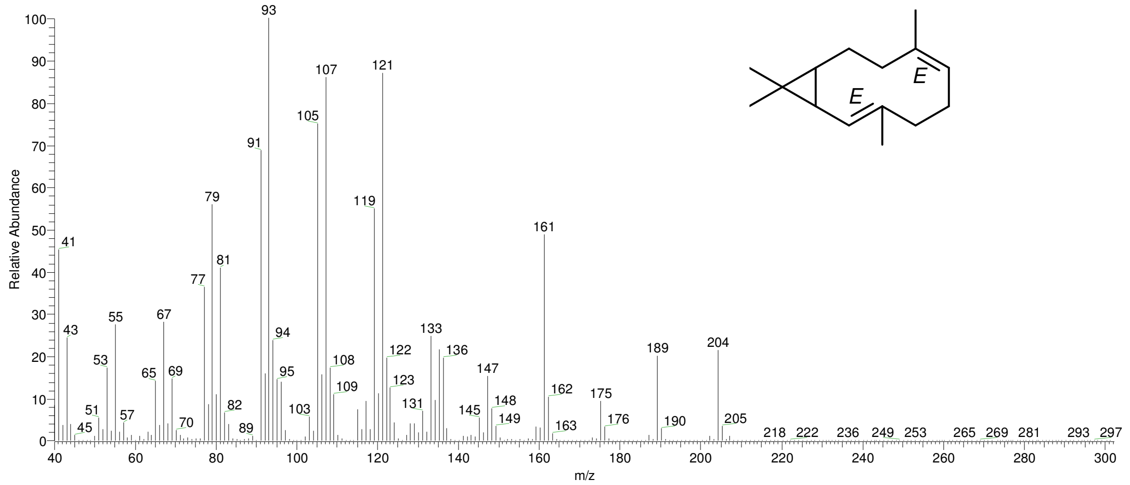

Figure S9. Mass spectrum of bicyclogermacrene (t_R 29.13 min).

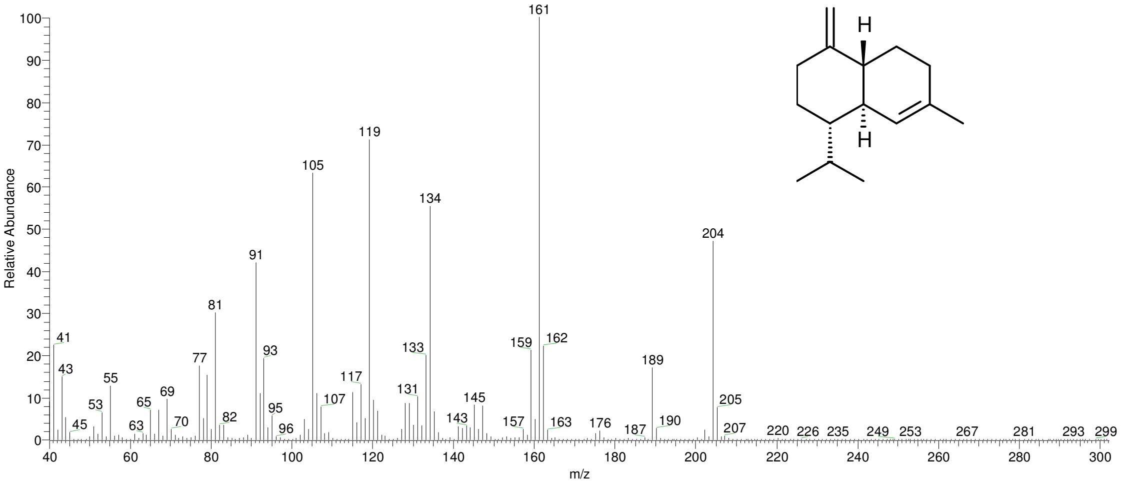

Figure S10. Mass spectrum of δ-cadinene (t_R 29.96 min).

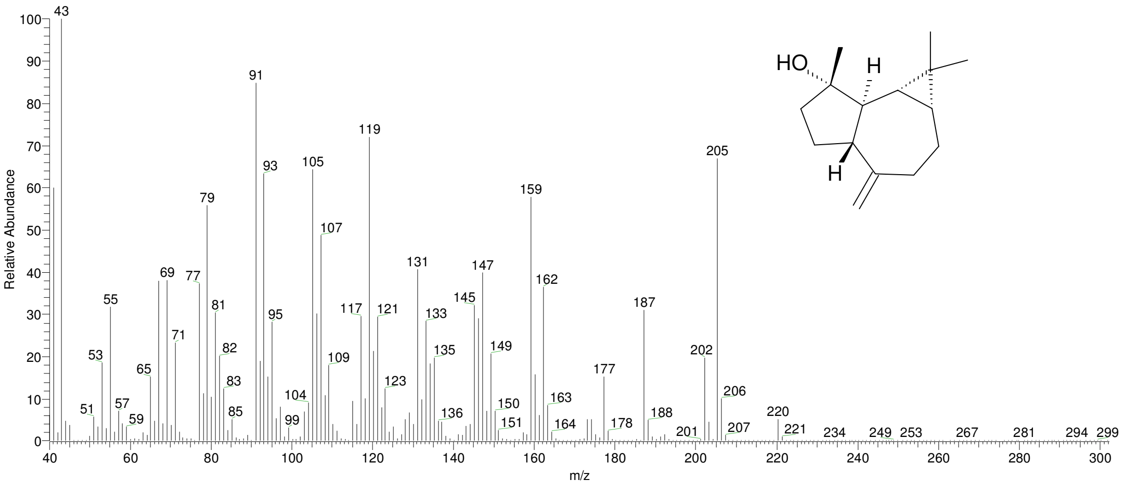

Figure S11. Mass spectrum of spathulenol (t_R 31.51 min).

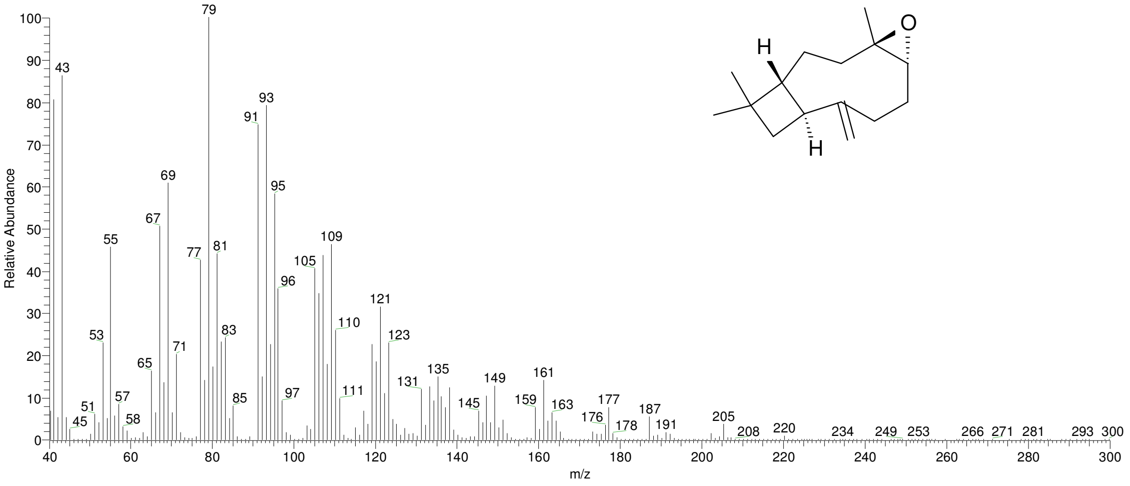

Figure S12. Mass spectrum of caryophyllene oxide (t_R 31.64 min).

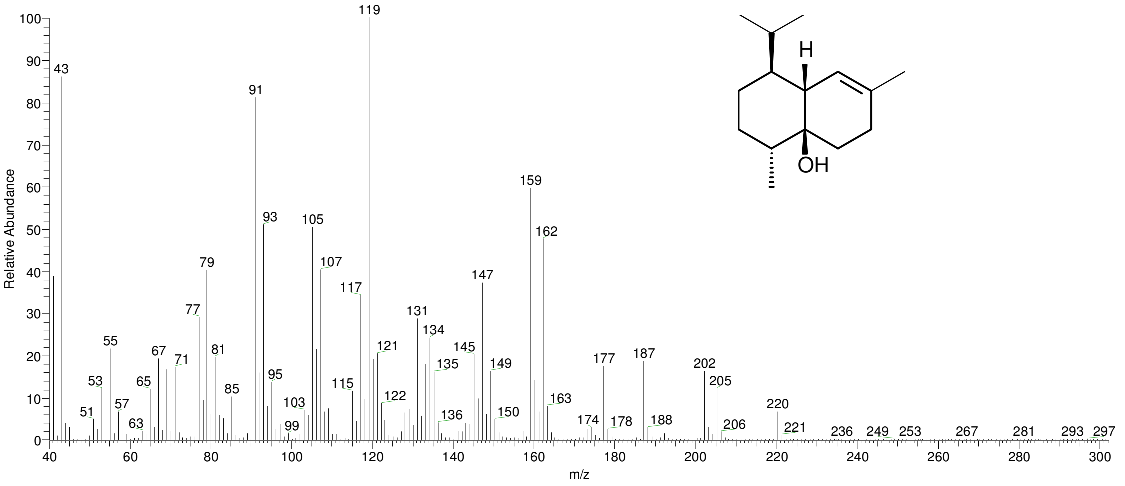

Figure S13. Mass spectrum of 1-*epi*-cubenol (t_R 32.98 min).

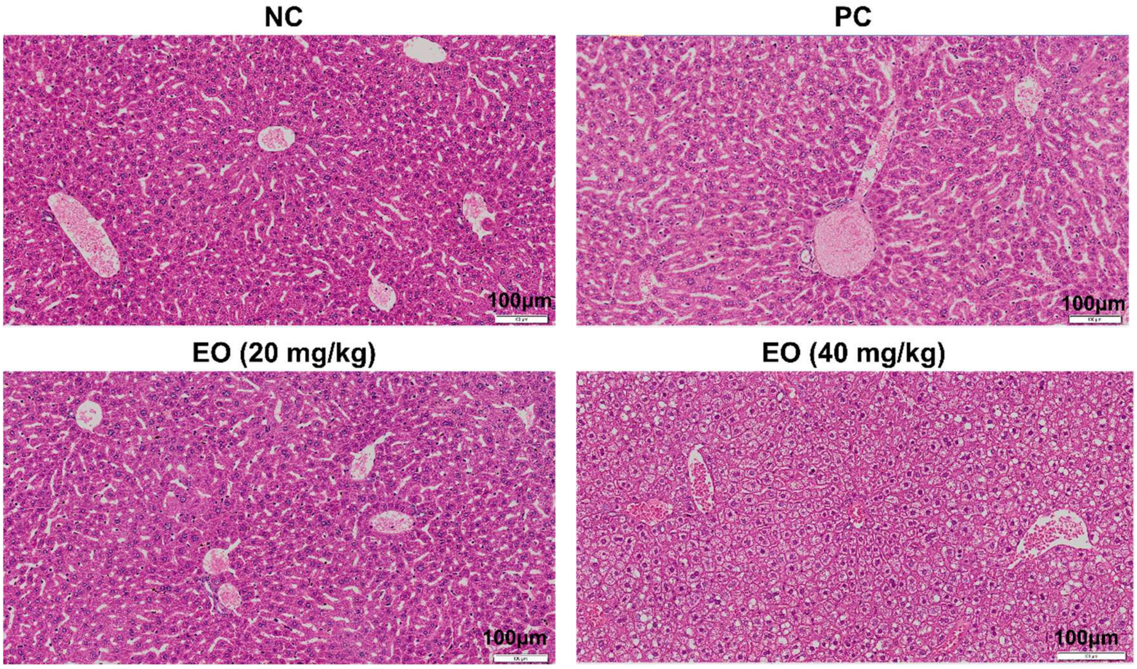

Figure S14. Representative photomicrographs of the livers of the C.B-17 SCID mice with HepgG2 cell xenografts treated with *G. olivacea* leaf EO. Negative control (**NC**) was treated with vehicle (5% DMSO) used for diluting EO, and doxorubicin (0.8 mg/kg) was used as positive control (**PC**).

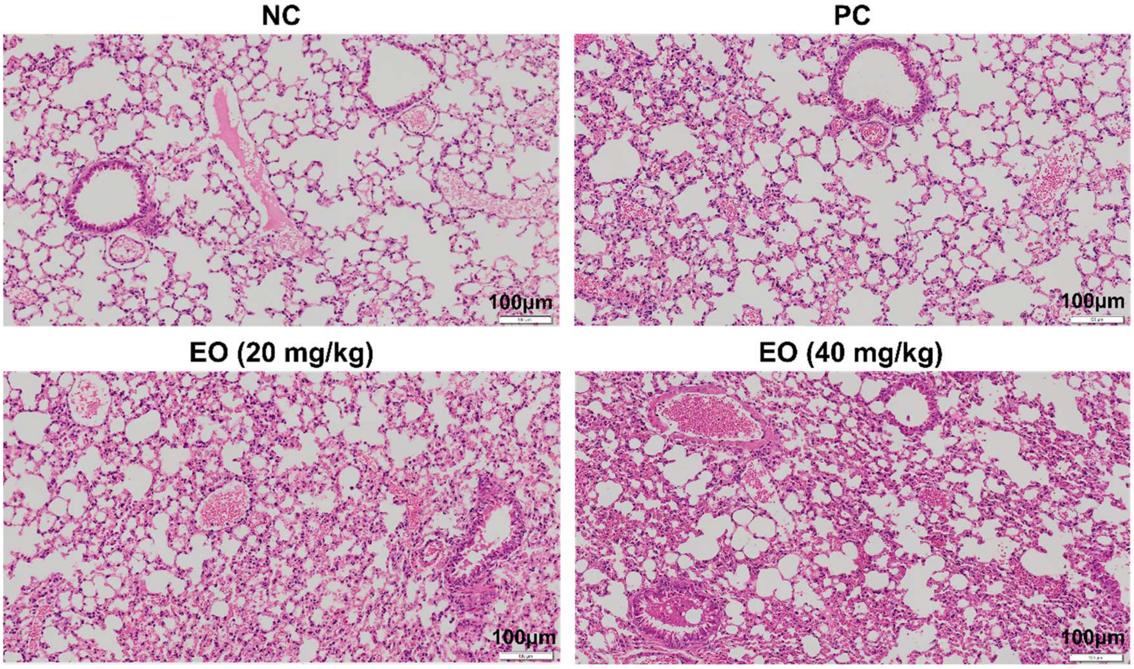

Figure S15. Representative photomicrographs of the lungs of the C.B-17 SCID mice with HepgG2 cell xenografts treated with *G. olivacea* leaf EO. Negative control (**NC**) was treated with vehicle (5% DMSO) used for diluting EO, and doxorubicin (0.8 mg/kg) was used as positive control (**PC**).

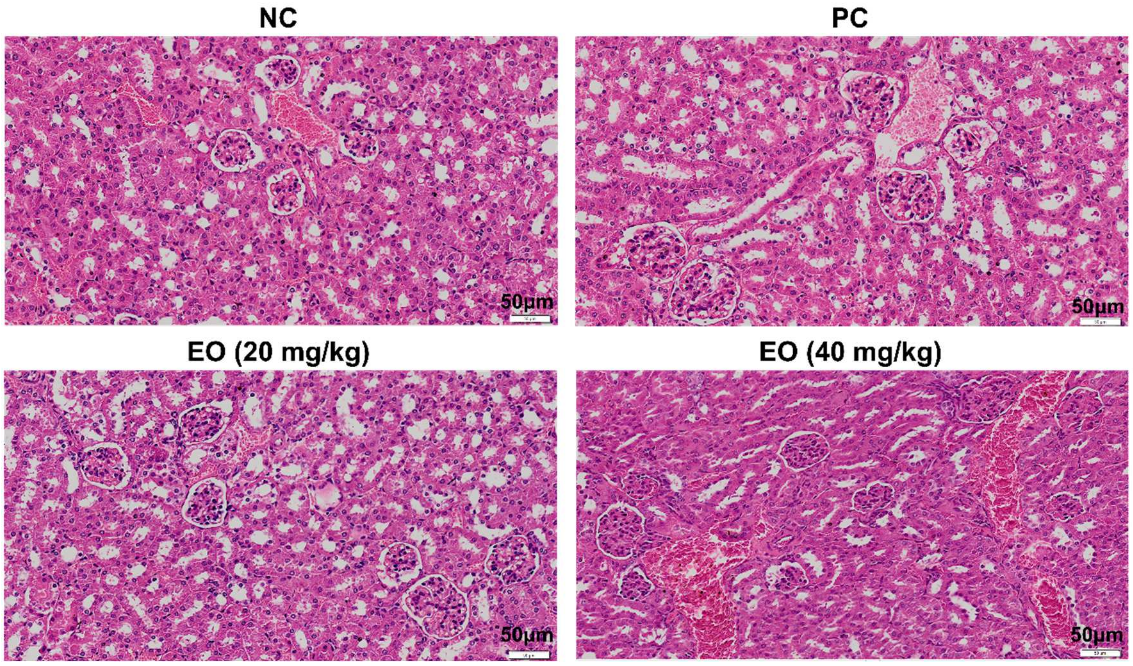

Figure S16. Representative photomicrographs of the kidneys of the C.B-17 SCID mice with HepgG2 cell xenografts treated with *G. olivacea* leaf EO. Negative control (**NC**) was treated with vehicle (5% DMSO) used for diluting EO, and doxorubicin (0.8 mg/kg) was used as positive control (**PC**).
